# Comorbid early esophageal cancer and *Gongylonema pulchrum* infection: a case report

**DOI:** 10.1186/s12876-021-01873-8

**Published:** 2021-07-31

**Authors:** Qiaozhi Zhou, Yongqiu Wei, Huihong Zhai, Shaogang Li, Rui Xu, Peng Li

**Affiliations:** 1 Department of Gastroenterology, Beijing Friendship Hospital, Capital Medical University, National Clinical Research Center for Digestive Disease, Yongan Road, Xicheng District, Beijing, 10050 China; 2 Beijing Tropical Medicine Research Institute, Beijing Friendship Hospital, Capital Medical University, Beijing, China; 3 Department of Pathology, Beijing Friendship Hospital, Capital Medical University, Beijing, China

**Keywords:** *Gongylonema pulchrum*, Esophageal squamous cell carcinoma, Carcinogenic effect, Parasites, Gongylonemiasis

## Abstract

**Background:**

*Gongylonema pulchrum* is a zoonotic parasite rarely found in humans. To date, there have been no reports on the carcinogenic properties of *G. pulchrum*, and there are few reports overall on the relationship between esophageal cancer and parasites.

**Case presentation:**

This report describes the first case of esophageal gongylonemiasis coexisting with early esophageal cancer. The patient had no high-risk factors for esophageal cancer, such as smoking, flushing after drinking, or tumor history. We speculate the existence of unknown links between esophageal cancer and parasitic infection in this patient.

**Discussion and conclusions:**

We report the first case of a human presenting both esophageal *G. pulchrum* infection and esophageal squamous cell carcinoma with the hope that it may provide evidence for a new hypothesis of tumorigenesis.

**Supplementary Information:**

The online version contains supplementary material available at 10.1186/s12876-021-01873-8.

## Background

The gullet worm *Gongylonema pulchrum* (Molin, 1857) is parasitic of the upper digestive tract of various mammals and birds worldwide, such as sheep, cattle, camels, donkeys, cervids, pigs, equids, bears, rodents, and primates [1, 2]. *Gongylonema pulchrum* is very rarely found in humans [3–6]. Indeed, fewer than 200 cases have been reported, with fewer than 10 cases involving infection of the esophagus, and no cases inducing serious symptoms [3].

Cancer may be induced by infectious agents, though the potential for infectious agents such as bacteria and viruses, including *Treponema denticola*, *Streptococcus anginosus*, human papillomavirus (HPV) and Epstein-Barr virus, as a cause of esophageal cancer remains unclear [7, 8]. To our knowledge, there are no reported human cases of comorbid esophageal cancer and *G. pulchrum* (parasite) infection [7]. Here, we report a rare case of a 59-year-old Chinese man who presented with esophageal *G. pulchrum* infection coexisting with early esophageal cancer (T1aN_0_M_0_).

## Case presentation

A 59-year-old man with complaints of epigastric discomfort for 5 months underwent upper endoscopy in a local hospital in July 2018; the gastroscopy report described a “foreign body in the mucosa of the esophagus”. At that time, a nematode body was removed under endoscopy, and parasitic infection was considered. The removed worm body was discarded without further detection. The patient was then referred to our hospital after approximately two weeks in August 2018, and gastroscopy was recommended again.

**Fig. 1:**
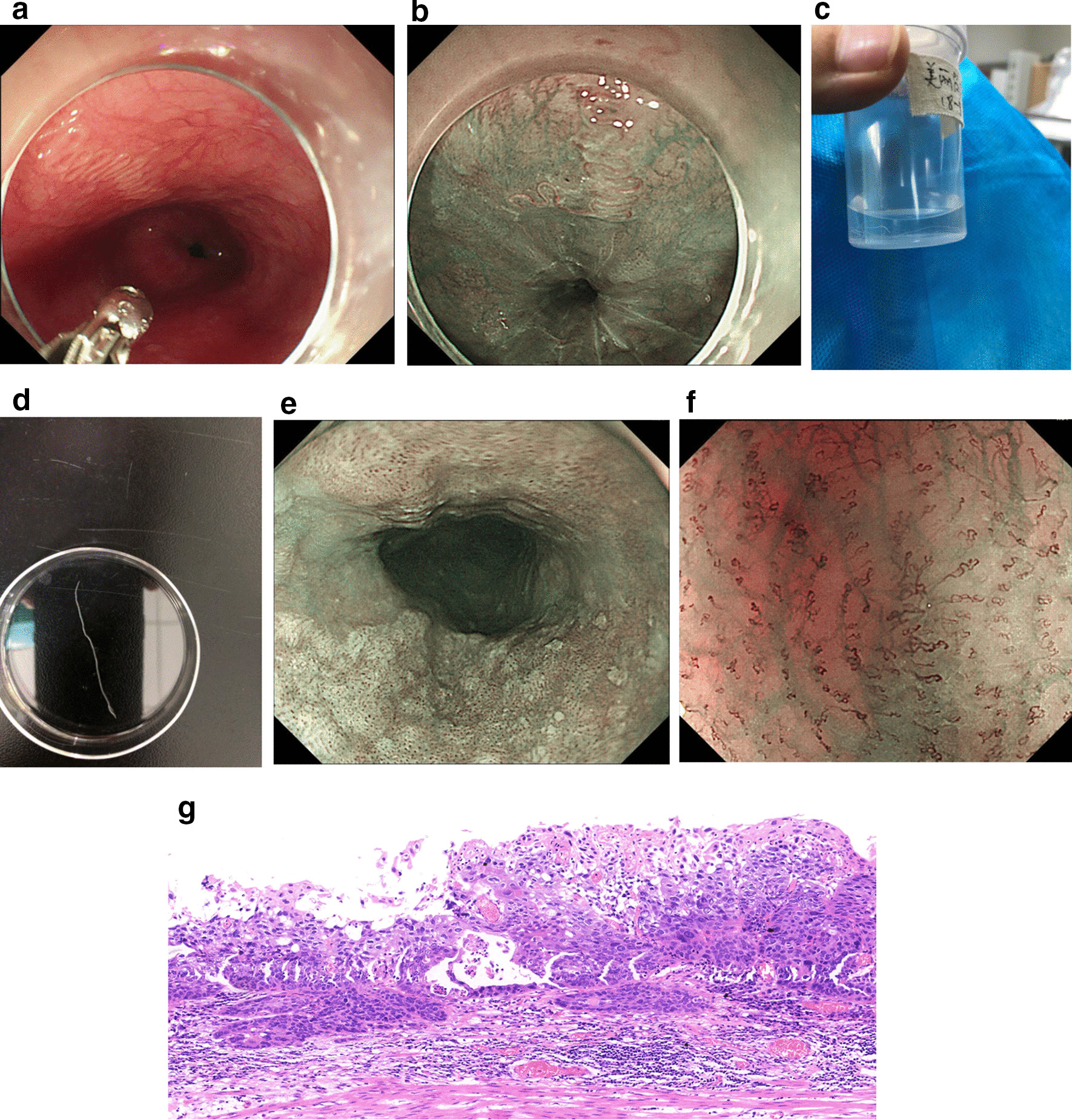
**a** Serpentine path of *Gongylonema pulchrum* in esophagus mucosa at 35 cm from the incisors in a 59-year-old-man with epigastric discomfort of 5 month’s duration. **b** The serpentine path of *Gongylonema pulchrum* viewed on narrow-band imaging. **c** The wormlike object was stored in formalin. **d** The wormlike object was displayed in a straight line. **e** A large area of rough surfaced esophageal mucosa with background coloration positive. **f** Observed under magnifying gastroscopy, most intraepillary capillary(IPCL) are type B1, and a few of them are type B2, suggesting an early esophageal squamous cell carcinoma. **g** Esophageal squamous cell carcinoma, islands and cords of malignant squamous cells, H&E

The endoscopy showed a white movable worm-like object adhering to the esophageal mucosa at approximately 35–38 cm from the incisors, which was more clearly depicted by narrow-band imaging (Fig. 1a, b). Observing the peristalsis of the worm, we were able to distinguish its head and tail, and the worm was carefully removed in one piece using biopsy forceps to grab the head and placed in formalin (Fig. 1c, d, Additional file 1). Parasitologists at our hospital confirmed that the object was a mature female *G. pulchrum* individual, which may have been living in the body for at least 1 year.

No other worms were found in the patient’s oral cavity, esophagus, stomach or duodenum. Physical examination and clinical examination revealed no abnormalities, and no parasite eggs were observed in multiple stool tests. This patient had no high-risk factors for esophageal cancer, such as smoking, flushing after drinking, or tumor history. He was a farmer in Hebei Province, China, and he had not traveled abroad. His living environment was relatively poor, and he also admitted to drinking unboiled water and eating uncooked vegetables that he grew with human feces fertilization. No animal was kept in his house. We suppose that the route of transmission was accidental ingestion of water or food contaminated by intermediate insect hosts.

**Fig. 2:**
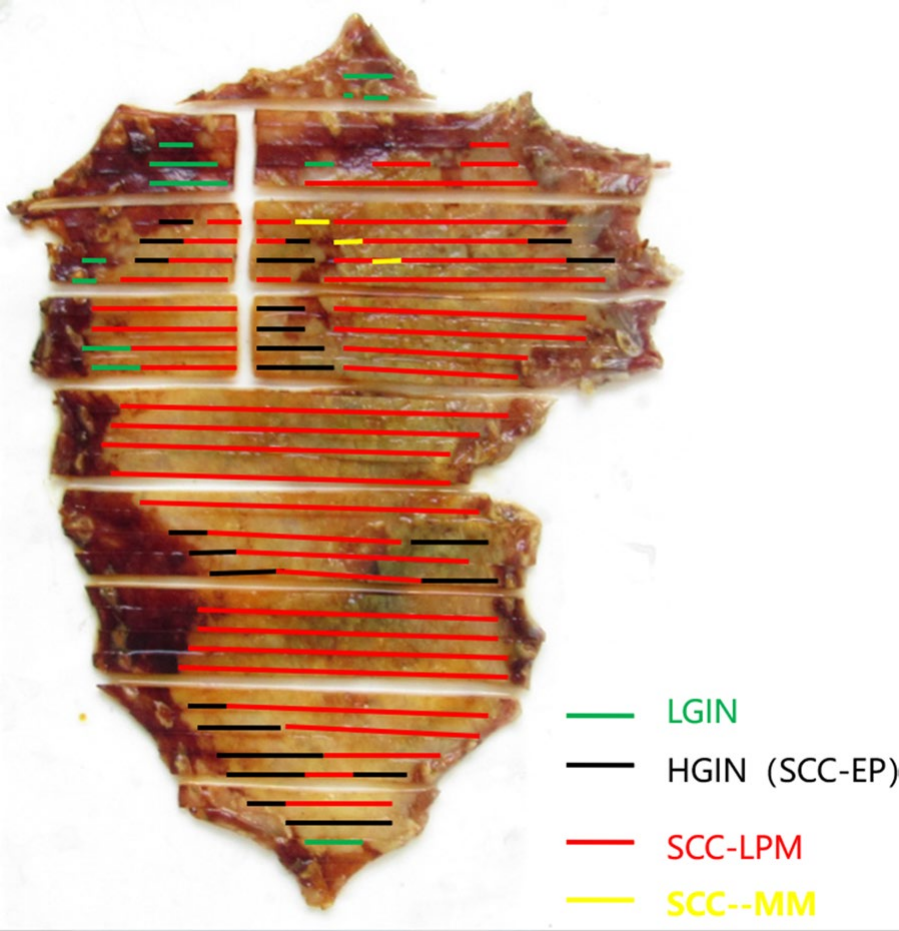
Pathological restoration of excised esophageal early cancer lesions. LGIN, low-grade intraepithelial neoplasia. HGIN, high-grade intraepithelial neoplasia. SCC, squamous cell carcinoma. EP, carcinoma in situ. LPM, tumor invasion to the lamina propria mucosae. MM, tumor invasion to the muscularis mucosa

In addition to the worm, this patient also had a large area of early esophageal cancer around the esophagus, which was 19–25 cm away from the incisors (Fig. 1e). Magnifying gastroscopy and ultrasound endoscopy indicated that the lesion was limited to the M_2_ layer (Fig. 1f), and no lymph node metastasis was found on enhanced computed tomography (CT). The patient underwent endoscopic submucosal dissection (Figs. 1g and 2). The pathological report revealed M_2_-infiltrated early esophageal squamous cell carcinoma of the esophagus, with focal infiltration of the muscularis mucosa but no complete infiltration. As no tumor tissue was present at the resection margin, curative resection was achieved. The squamous cell carcinoma lesion did not contain any parasites.

Forty-eight hours after the operation, the patient began taking prednisone acetate 30 mg Qd to prevent stenosis and albendazole for 7 days as antiparasitic treatment. The patient developed esophageal stenosis after surgery, though it steadily improved after multiple dilations. There was no parasite recurrence after repeated gastroscopy, and the patient’s symptoms disappeared.

## Discussion and conclusions

The gullet worm *G. pulchrum* (Molin, 1857) is a parasite in the upper digestive tract of various mammals around the world and does not normally infect humans. In contrast, *G. pulchrum* infections commonly occur in ruminants, such as domestic cattle, which are a suitable definitive host; infection has also been reported in equids, bears, skunks, swine, primates, rodents, rabbits, hedgehogs, and birds [3]. These animals usually acquire parasite infestations by feeding on insects (the intermediate host), which may fall into water or are crushed on vegetables. The infective larvae burrow into the mucosa and submucosa of the stomach or duodenum of the host and migrate to the oral cavity or esophagus after approximately 60–80 days of development. Mature nematodes can live in the body for 1 year or even more than 10 years [3].

As mentioned above, this infection rarely occurs in humans, with fewer than 200 cases since the first case of human gongylonemiasis was reported in 1850 [3, 9]. In humans, these worms are often found in the oral cavity, where they mostly cause the sensation of a migrating thread-like form, irritation, minor aches, and nausea, with no lesions noticed [10]. It is extremely rare for worms to be located in the esophagus. To the best of our knowledge, fewer than 10 cases of esophageal infection have been reported worldwide [11–14], usually accompanied by symptoms of local irritation, such as foreign-body sensation, minor aches, nausea and cough. There are no reports of tumors with gongylonemiasis in humans. Therefore, this report is the first case of esophageal *G. pulchrum* infection combined with esophageal squamous cell carcinoma in humans.

Cancer may be induced by some complex microenvironmental and physical conditions, and infection, such as with viruses and bacteria, appears to be one of the most important causes[7, 8, 15]. In fact, associations between parasite infection and human cancer have been well evidenced for years, such as schistosomiasis for urinary bladder cancer, colorectal cancer, hepatocellular carcinoma, squamous cell carcinoma, adenocarcinoma, opisthorchiasis for cholangiocarcinoma, clonorchiasis for cholangiocarcinoma, and strongyloidiasis for human T-lymphotropic virus type-1 (HTLV-1)-induced lymphomas/leukemias and colon adenocarcinoma[16–18]. The proposed mechanism of carcinogenesis is chronic inflammation, oxidative stress caused by parasite-derived molecules, cell proliferation, stimulation of HTLV-1 replication and oligoclonal expansion of HTLV-1-infected lymphocytes [7, 8].

With regard to esophageal cancer, an infectious cause remains unknown, though it is possible. There are several types of bacteria and viruses associated with esophageal cancer, such as *T. denticola*, *S. anginosus*, human papillomavirus (HPV), Epstein-Barr virus, and polyoma viruses [11]. Although rare cases of parasites in the esophagus have been reported, none have been linked to human esophageal cancer. Nevertheless, in recent studies, *Spirocerca lupi*, a parasitic nematode of canids, was found to play a possible role in immunomodulation in the induction of esophageal neoplastic transformation in dogs [12, 19]. In our case, the fact that no parasites were detected inside the tumor may be based on poor living conditions for nematodes inside a tumor compared with unaltered esophageal epithelia. Further study is needed to determine the parasite’s preferred habitat.

An animal case report described a 17-year-old female vari (*Lemur macaco* variegates; KEHR 1792) with both esophageal *G. pulchrum* infection and esophageal squamous cell carcinoma [20]. We report for the first time coexistence of esophageal *G. pulchrum* infection and esophageal squamous cell carcinoma in a human. In general, it is difficult to identify pathogens as causative agents of cancer, owing to the usually long latency between primary infection and cancer development, and the causal involvement of parasitic worms in human cancer is typically first suspected on the basis of larger epidemiological data.

Although our patient did not appear to have any risk factors associated with esophageal cancer, the association between esophageal *G. pulchrum* infection and early esophageal cancer seen in this case may be incidental because it appears to be the first human case and the second mammalian case reported. Nevertheless, it is important to report this case for determination if this is only a casual association or there exists a predisposition for this type of cancer. Thus far, there have been fewer than 10 cases of esophageal *G. pulchrum* infection reported in the literature.

Despite a lack of definitive proof for a pathogenic relationship between *G. pulchrum* infection and esophageal cancer, this case provides evidence for a new hypothesis of tumorigenesis.

## Supplementary Information

**Additional file 1**. The process of removing the Gongylonema pulchrum under endoscopy.

## Data Availability

The datasets generated and analyzed during the current study are available from the corresponding author upon reasonable request.

## Abbreviations

HPV: Human papillomavirus
CT: Computed tomography
HTLV-1: Human T-lymphotropic virus type-1
